# Evaluation of the Effect of Lime Fruit Juice on the Anticoagulant Effect of Warfarin

**DOI:** 10.4103/0975-1483.66808

**Published:** 2010

**Authors:** GKA Adepoju, T Adeyemi

**Affiliations:** Department of Clinical Pharmacy and Biopharmacy, Faculty of Pharmacy, Olabisi Onabanjo University, Sagamu, Ogun State, Nigeria

**Keywords:** Anticoagulant, complementary and alternative medicines, interaction, lime fruit, therapeutic effectiveness, traditional medicine, warfarin

## Abstract

**Aim:**

*Citrus aurantifolia* (Family Rutaceae) is commonly known as a familiar food and medicine, and s therapeutic effectiveness in a variety of diseases has been suggested in traditional medicine. Various complementary and alternative medicines (CAM) have been shown to interact with orthodox medicines. Hence, the aim of this study is to investigate such a phenomenon particularly the interaction of lime fruit juice with warfarin.

**Materials and Method:**

Wistar strain albino rats of both sexes weighing between 190 and 230g were administered with oral doses of the respective drugs used depending on the groups of animals. Effects on the anticoagulant activity of warfarin were determined by standard laboratory methods.

**Result:**

Lime fruit juice caused a reduction in the anticoagulant activity of warfarin.

**Conclusion:**

This finding has shown that CAM can interact with orthodox medicines hence, warfarin prescribers need to be aware of the usage of CAM and monitor the international normalized ratio (INR) of their patients more frequently.

## INTRODUCTION

*Citrus aurantifolia* (Lime) is commonly known as familiar food and medicine, and its therapeutic effectiveness in a variety of diseases has been suggested in traditional medicine.[1–3] The juice of citrus fruit shows its actions as a cytotoxic;[4] and an antimicrobial against upper respiratory tract bacterial pathogens. Adeleye and Opiah[5] Gharagozloo *et al*.[6] documented the anti-proliferative activity of concentrated lime fruit juice extract on lymphoblastoid cell line. In addition, they reported the immunomodulatory effects on activated human lymphocytes, likely due to the protein components of the extract.[7]

The root of *C. aurantifolia* is used in traditional medicine for the treatment of fever. The wide range of bioactive compounds from the citrus species has been found to possess anti-infection and anti-inflammatory activities.[8,9] Guthrie and Carroll, Hollman, Kawaii, Lam and Hasegawa[10–13] showed that the flavonoids, limonoids, and ascorbic acid are groups of citrus phytochemicals and micronutrients which are responsible for the anti-inflammatory and antitumor activities. The citrus limonoids inhibit the chemically induced colon carcinoma in addition to the potential use of citrus flavonoids in cancer treatment.[14–16]

Chunlarathanaphorn *et al*.[3] have shown that the water extract from the root of *C. aurantifolia* administered orally did not cause acute or sub-chronic toxicities in male or female rats. A large proportion of patients use herbal remedies with a potential to interact with prescribed drugs. This can be dangerous particularly if the therapeutic window of the prescribed drug is small, as with warfarin.[17] Many complementary medicines have confirmed potential interactions with warfarin. Myers[18] and Elmer[19] showed that there were potential interactions between complementary/alternative medicines and conventional medicines. Various authors have shown specific interactions of herbal medicines (complementary medicines)[17–21] Others showed the possible interaction between warfarin and cranberry juice.[22] Interactions with warfarin can lead to either an increased or decreased anticoagulant effect. Those that increase the anticoagulant effect significantly increase the risk of serious hemorrhage. Interactions that decrease the anticoagulant effect significantly increase the risk of thrombo-embolic complications of the condition for which warfarin was pressured.[18] The effect of grapefruit juice on the pharmacokinetics and pharmacodynamics of the calcium antagonist amlodipine was assessed[23] just as the effects of its long-term ingestion on nifedipine were studied by Mohri *et al*.[24]

As lime fruit juice is commonly used as food and medicine, its potential interactions with orthodox complementary alternative products have not been studied. Hence, this study aimed at assessing the potential interaction between lime fruit juice and warfarin.

## MATERIALS AND METHODS

### Chemicals

Normal Saline, Warfarin sodium (Norton Health Ltd, Essex, England). *Citrus aurantifolia* has its identification confirmed at the Forestry Research Institute of Nigeria (FRIN), Nigeria.

### Animal and experimental design

Wistar strain albino rats of both sexes weighing between 190 and 230g were used for the experimental study. They were obtained from the Animal House, University of Ibadan, Nigeria. The animals have not been involved in any experimental design before. They were kept under standard laboratory environmental conditions and were fed with standard rat pellet (feed) and pure drinking (potable) water ad libitum for 21 days before the start of the experiment.

The rats were randomly divided into five groups of five rats per group for the experimentation. Group 1 animals served as the control group and were administered with oral doses of 0.5 ml of normal saline everyday for 14 days. Group II rats were given a single oral dose of 0.5 ml of 1:1 dilution of line juice everyday for 14 days, while Group III received a single oral dose of 0.5 ml of 1:2 dilution of lime fruit juice everyday for 14 days.

Group IV rats received a single oral dose of 0.5 ml normal saline (0.9% NaCl) everyday for 14 days. On the 14^th^ day, they were administered a single oral dose of warfarin (1.5 mg/kg body weight). Group V animals were administered a single oral dose of 0.5 ml of 1:1 dilution of lime fruit juice everyday for 14 days. On the 14th day, the animals were administered a single oral dose of warfarin (1.5 mg/kg body weight). All the doses were administered using an oral cannula.

Blood samples were obtained from the animals through decapitation 24 h after the last administration of the respective group doses. The blood samples were collected in heparinized tubes. Effects on the hematological parameters were determined by the standard methods of Schimit, Trenk and Jahncler;[25] Toro and Ackermann[26] Ivy *et al*.[27] was adopted to determine the bleeding time. The consent of the Medical Ethics Committee of the Medical College was obtained before the commencement of the study.

### Statistical analysis

This was carried out using the Student’s t-test comparing the control (Group 1) with the treated groups (Groups II-V) at *P*<0.05. The result values were expressed as mean ± SEM (standard error of the mean).

## RESULT AND DISCUSSION

The decrease in bleeding time observed in Groups II and III showed that lime fruit juice has hemostatic effect; however, the decrease was not statistically significant. There was no change in bleeding time upon the administration of warfarin (Group IV) compared to the control group (Group I). However, lime juice administered together with warfarin (Group V) significantly reduced the bleeding time. Bleeding time is a measure of the primary hemostasis where-in platelets “plugs” are formed by collagen-induced aggregation of platelets and thrombin-induced formation of fibrin.

Hence, lime alone (Groups II and III) did not significantly decrease the bleeding time, but it significantly reduced the bleeding in the presence of warfarin. Hence, lime fruit juice exerted an antagonistic hemostatic effect on the anticoagulant hematological activity of warfarin. Rather than increase the bleeding time, lime fruit juice decreased the bleeding time.

Warfarin alone (Group IV) reduced the red blood cell count (RBC) [Table 1] when compared to the control (Group I) and the other groups. However, this was not statistically significant. Hence, there were no statistically significant changes in the values of the PCV for all the groups. The reduction in RBC by warfarin contributed to its anticoagulant effect. This was because the number of cells to be trapped in the “clotting mesh” was reduced. This in addition to the prolongation of the bleeding time potentiated the anticoagulant effect of warfarin [Table 1].

**Table 1:** Showing the effects of Lime on the anticoagulant activity of Warfarin

Group	Durgs	RBC count/mm^3^	PCV %	Platelet count /mm^3^	PT count /mm^3^	BT (min-1)
I	Control	4.34 ± 0.094	38 ± 0.71	183.6 ± 8969.82	8.74 ± 0.22	253.2 ± 5.16
II	Lime (1:1 dil.)	4.20 ± 0.12	39.6 ± 0.81	171.0 ± 6715.56	8.58 ± 0.24	243.6 ± 7.25
III	Lime (1:2 dil.)	4.12 ± 0.073	37.8 ± 0.66	190.6 ± 6423.2	9.42 ± 0.21	241.2 ± 8.36
IV	Warfarin	4.04 ± 0.011	39.6 ± 0.40	169.0 ± 5639.1	9.0 ± 0.3	253.2 ± 5.16
V	Lime (1:1 dil) + Warfarin	4.06 ± 0.011	37.2 ± 0.38	167.4 ± 5836.1	6.88 ± 0.32*	137.6 ± 7.98*

The mean platelet count of Group V animals decreased compared to the control and other groups. However, this was not statistically significant. Hence, lime fruit juice reduced the anticoagulant activity of warfarin though not statistically significant.

The co-administration of lime fruit juice to warfarin reduced the prothrombin time. This was statistically significant when compared to the control and Group IV. Hence, the lime fruit juice significantly reduced the warfarin effect on prothrombin time. Ironically, lime fruit alone increased the prothrombin time significantly more than warfarin alone (Group III). Juxtaposingly was it warfarin that reduced the prothrombin time effect of lime fruit juice?; However each antagonized the activity of the other on prothrombin time. This was statistically significant.

## CONCLUSION

Lime fruit consumption causes a reduction in the anti-coagulant activity of warfarin. This finding has shown that lime fruit juice can adversely interact with other medications. Hence, warfarin prescribers need to be aware of the usage of complimentary medicines and monitor the international normalized ratio (INR) more frequently. As a precaution, patients on warfarin should have their INR measurements about 2 and 7 days after starting or changing any herbal treatment.[2]
